# Ketogenic diet in treating sepsis-related acquired weakness: is it friend or foe?

**DOI:** 10.3389/fnut.2024.1484856

**Published:** 2024-11-28

**Authors:** Yanmei Miao, Leiyu Xie, Shaolin Chen, Xiaoming Zhang, Wenjie Liu, Peng Xie

**Affiliations:** ^1^Department of Critical Care Medicine of the Third Affiliated Hospital (The First People's Hospital of Zunyi), Zunyi Medical University, Zunyi, China; ^2^Department of Nursing of Affiliated Hospital, Zunyi Medical University, Zunyi, China; ^3^Department of Molecular and Cellular Biology, Baylor College of Medicine, Houston, TX, United States; ^4^Department of Anesthesiology, The Second Affiliated Hospital, Hengyang Medical School, University of South China, Hengyang, China; ^5^Department of Critical Care Medicine, The Second Affiliated Hospital, Hengyang Medical School, University of South China, Hengyang, China

**Keywords:** gut microbiota, ICU-acquired weakness, ketone bodies, ketogenic diet, sepsis-related acquired weakness

## Abstract

**Background:**

Sepsis is the body’s extreme response to an infection leading to organ dysfunction. Sepsis-related acquired weakness (SAW), a critical illness closely related to metabolic disorders, is characterized by generalized sepsis-induced skeletal muscle weakness, mainly manifesting as symmetrical atrophy of respiratory and limb muscles. Muscle accounts for 40% of the body’s total mass and is one of the major sites of glucose and energy absorption. Diet affects skeletal muscle metabolism, which further impacts physiology and signaling pathways. The ketogenic diet (KD) is a high-fat, low-carbohydrate diet that has shown benefits in patients with a variety of neuromuscular disorders. Patients with SAW are in a hypermetabolic state and can consume approximately 1% of total body muscle mass in a day. Due to the decreased total body energy expenditure secondary to starvation, skeletal muscles enter a low metabolic state, with reduced gluconeogenesis and protein consumption and elevated levels of ketone bodies. The latest research suggests that KD may be a new strategy for SAW prevention and treatment, but its mechanism is still unclear.

**Objective:**

Our article aims to explore the effect and mechanism of KD on SAW. And we hope that our review will inspire further research on the KD and foster the exploration of novel strategies for combating SAW.

**Methods:**

Search medical databases and related academic websites, using keywords such as “Sepsis-related acquired weakness,” “ketogenic diet,” and “skeletal muscle,” and select representative literature. Using the method of induction and summary, analyze the effect and mechanism of KD on SAW.

**Results:**

Compared with early nutrition, KD has a more protective effect on SAW, but its mechanism is complex. Firstly, KD can alter energy metabolism substrates to affect SAW’s energy metabolism; Secondly, KD can directly act as a signaling molecule to improve mitochondrial function in skeletal muscle and stimulate skeletal muscle regeneration signaling molecules; Thirdly, KD can affect the gut microbiota to exert anti-inflammatory effects, enhance immunity, and thus protect SAW.

**Conclusion:**

KD has a protective effect on SAW, which includes improving energy metabolism, stimulating muscle regeneration signals, optimizing gut microbiota composition, and reducing inflammation and oxidative stress.

## Introduction

ICU-acquired weakness (ICU-AW) is a neuromuscular dysfunction that has no other reasonable etiology besides critical illness and its treatment (1). ICU-acquired weakness is typically generalized, symmetrical, and affects limb (proximal more than distal) and respiratory muscles, whereas facial and ocular muscles are spared (2). According to statistics, globally, about 13–20 million patients receive life-supporting treatment in ICUs every year, and more than 1 million critically ill patients experience ICU-AW each year (3). Comorbid sepsis, prolonged motor inhibition, the application of mechanical ventilation, and malnutrition are risk factors for ICU-AW in critically ill patients (4), among which sepsis is an independent risk factor for ICU-AW. Up to 60–100% of patients with sepsis develop ICU-AW, which is known as sepsis-related acquired weakness (SAW) (5). SAW is a critical illness with unique metabolisms (6), for which there has been no effective clinical treatment (6). It often results in prolonged mechanical ventilatory support and hospitalization, increasing patient mortality and seriously affecting patients’ quality of life after discharge (7). Therefore, there is an urgent need for effective measures to prevent the occurrence and development of SAW, and to improve the survival rate and quality of life of critically ill patients.

Muscle accounts for 40% of the body’s total mass and is the primary site of glucose and energy absorption. For organisms that have high energy requirements, sufficient adenosine triphosphate (ATP) production is essential for muscle contraction and the maintenance of muscle function (8). Variations in diets affect not only muscle metabolism (9) but also signaling pathways for growth, survival, and other functions in skeletal muscle cells. Metabolic flexibility in response to a variety of stimuli, including dietary changes, is an important feature of muscle energetics. Changes in dietary metabolism impact muscle tissues not only through energy supply but also through metabolic intermediates. Specific changes in metabolism, such as activation of ketogenic metabolism, may have protective effects against SAW (10).

The ketogenic diet (KD) is formulated with a high percentage of fats, a low percentage of carbohydrates, and adequate proteins and other nutrients. It has been used for more than 100 years since its initial introduction in 1921 by Dr. Wilder at the Mayo Clinic (11). The KD, which was originally used for controlling epilepsy, especially hard-to-control epilepsy (12), is now being used in the management of obesity (13), polycystic ovary syndrome (14), cancer (15), diabetes mellitus (16), and traumatic brain injury (17), as well as amyotrophic lateral sclerosis (18), mitochondrial myopathies (19), primary sarcopenia (20), and other pathologic conditions (21). Recent studies have demonstrated that the KD may be a novel strategy for the treatment of SAW (22), but the mechanism is still unclear. In addition, KD has great plasticity in the prevention and treatment of muscle atrophy, but a lot of work is needed to clarify the overall situation. Therefore, this review will review the role and mechanism of KD in SAW, providing new methods and avenues for the prevention or treatment of SAW.

## The KD and early nutritional support

### The KD

**Figure 1:**
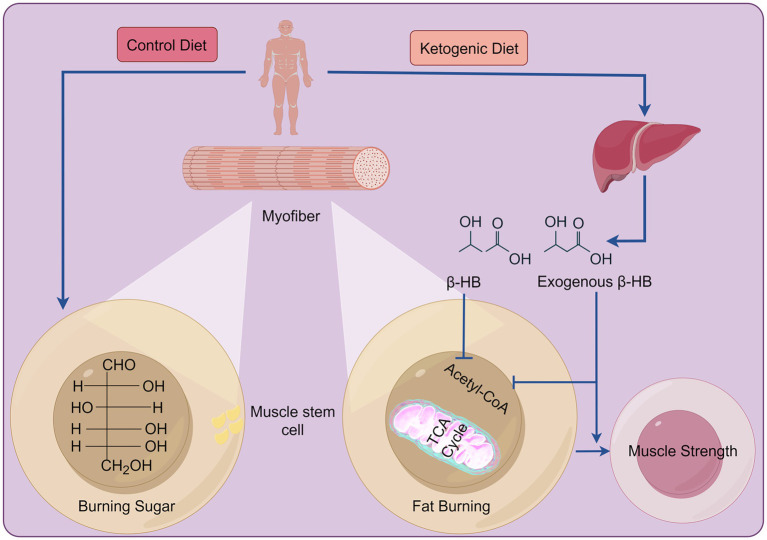
The differences between the KD and a standard diet in energy production. In a standard diet, carbohydrates serve as the primary energy source post-consumption. In contrast, the KD mainly relies on KBs for energy production. These ingested KBs, alongside the ones produced by the liver, enter the tricarboxylic acid cycle to generate energy. Created with Figdraw.com.

The main types of KDs include the classic KD, the medium-chain triglyceride KD, the modified Atkins diet, and the low-blood-sugar KD. The classic KD: Fat accounts for 70–75% of total calories, protein accounts for 20–25%, carbohydrates account for about 5%. Strictly controlling the intake of carbohydrates can promote the body to enter a ketotic state, relying on fat metabolism to produce ketone bodies for energy supply, which can effectively control epileptic seizures, especially for children with refractory epilepsy; The medium-chain triglyceride KD: emphasizes increasing the intake of medium chain triglycerides (MCT), which can be quickly absorbed by the body and converted into ketone bodies. Compared to ordinary fats, MCT is more likely to produce ketone bodies, making it more suitable for people who need to quickly enter a ketogenic state, such as patients with certain neurological diseases; The modified Atkins diet: Compared to the classic ketogenic diet, the restrictions on carbohydrates are relatively relaxed, allowing for a daily intake of 10–20 grams of net carbohydrates. The fat ratio is relatively reduced, and the protein ratio can be appropriately increased. This is suitable for epilepsy patients who are unwilling to accept a strict ketogenic diet and may also have certain benefits in weight loss and improving metabolic indicators; The low-blood-sugar KD: Pay attention to the glycemic index (GI) of food, choose low GI foods with a balanced ratio of fat and protein, which helps stabilize blood sugar levels, reduce blood sugar fluctuations, and improve insulin sensitivity to some extent (23). After metabolizing the KD *in vivo*, the levels of ketone bodies (KBs) (i.e., acetoacetic acid, *β*-hydroxybutyric acid [β-HB], and acetone) and fatty acids significantly increase. The synthesis of KBs, also known as ketogenesis, predominantly occurs in hepatocytes and less in astrocytes or renal cells (24). KB utilization occurs in the heart, skeletal muscles, and brain (Figure 1) (25). When there is a deficiency or decrease in dietary carbohydrates, the plasma insulin level decreases while the glucagon level increases, thereby promoting hepatic glycogenolysis and gluconeogenesis, as well as lipolysis in adipose tissue, which is mediated by hormone-sensitive lipase. Restricting carbohydrate intake for 4–7 days leads to failed glycogenolysis and increased ketogenesis with elevated levels of free fatty acids (FFA), acetyl coenzyme A (acetyl-CoA), and KBs (26). When glucose levels are low or carbohydrate consumption is insufficient, KBs become the primary source of energy and mediate cell signaling, post-translational modifications, inflammation, oxidative stress, and the synthesis of lipids like myelin and cholesterol (27).

### Early nutritional support

Inflammatory mediators can potently induce catabolism during sepsis and play a key role in catabolizing muscle proteins and driving lipolysis in adipocytes. Endogenous proteolysis associated with inflammation in skeletal muscles can rapidly progress to SAW (28). Early nutrition for critically ill patients refers to providing nutrition to patients in the early stages (within 48 h) after the onset of critical illness (29). Traditionally, providing patients with 80% full-calorie and protein (1.2–2.0 g/kg/d) feeds during the first week in the ICU optimizes their outcomes (30). However, the recent CALORIES and NUTRIREA-2 trials have shown that early nutritional support (enteral versus parenteral nutrition) does not alter the survival rate (31), and intentional undernutrition (low-volume enteral nutrition) in the first week of ICU stay has the same effects as early nutritional support (32). In addition, early nutritional support may create an extra metabolic burden, leading not only to higher blood glucose levels, aggravated infections (33), and adverse gastrointestinal reactions, but also to increased mechanical ventilation dependence, prolonged ICU stays, and hindered recovery from SAW (34). Overall, these studies have shown that early nutritional support in SAW is ineffective and may even cause harm (Table 1).

**Table 1:** Summary of literature review on early nutrition.

Author(s)	Title	Study aim	Year	Area
Reid CL, et al.	Muscle wasting and energy balance in critical illness	Determine whether muscle ultrasound is suitable for a larger ICU population (*n* = 50) (28)	2004	Britain
Reintam BA, et al.	Early enteral nutrition in critically ill patients: ESICM clinical practice guidelines	To provide evidence-based guidelines for early enteral nutrition (EEN) during critical illness (29)	2017	Switzerland
Heyland DK, et al.	Optimal amount of calories for critically ill patients: depends on how you slice the cake!	To examine the relationship between the amount of calories administered and mortality (*n* = 7,872) (30)	2011	Canada
Harvey SE, et al.	Trial of the route of early nutritional support in critically ill adults	Exploring the most effective ways to provide early nutritional support for critically ill adult patients (*n* = 2,400) (31)	2014	Britain
Rice TW, et al.	Randomized trial of initial trophic versus full-energy enteral nutrition in mechanically ventilated patients with acute respiratory failure	Testing the initial low volume (i.e., nutritional) enteral nutrition can reduce the occurrence of gastrointestinal intolerance/complications and improve the outcome of initial full energy enteral nutrition in patients with acute respiratory failure (*n* = 200) (32)	2011	America
Heidegger CP, et al.	Optimization of energy provision with supplemental parenteral nutrition in critically ill patients: a randomized controlled clinical trial	Evaluating whether using EN plus parenteral nutrition (SPN) to deliver 100% energy target on ICU days 4–8 can optimize clinical outcomes (*n* = 153) (33)	2013	Switzerland
Reintam Blaser A, et al.	How to avoid harm with feeding critically ill patients: a synthesis of viewpoints of a basic scientist, dietitian and intensivist	It is recommend to recommend the best feeding strategy for critically ill patients (34)	2023	Switzerland
Weijs PJ, et al.	Early high protein intake is associated with low mortality and energy overfeeding with high mortality in non-septic mechanically ventilated critically ill patients	Early protein and energy feeding in critically ill patients is heavily debated (*n* = 843) (35)	2014	the Netherlands
Lee ZY, et al.	The effect of higher versus lower protein delivery in critically ill patients: a systematic review and meta-analysis of randomized controlled trials	To compare the effect of higher versus lower protein delivery (with similar energy delivery between groups) on clinical and patient-centered outcomes in critically ill patients (*n* = 1,731) (36)	2021	Canada
Weijs PJ, et al.	Optimal protein and energy nutrition decreases mortality in mechanically ventilated, critically ill patients: a prospective observational cohort study	Exploring mechanical ventilation and optimal nutritional therapy for critically ill patients (*n* = 886) (37)	2012	Austria
Lindner G, et al.	Hypernatremia in the critically ill is an independent risk factor for mortality	Assessing the prevalence of hypernatremia and its impact on mortality and ICU length of stay (LOS) (*n* = 981) (38)	2007	Austria
Gunst J, et al.	Amino acid supplements in critically ill patients	Taking single amino acid supplements during critical illness and administering glutamine may be harmful (39)	2018	Belgium
Li F, et al.	Role of TFEB Mediated Autophagy, Oxidative Stress, Inflammation, and Cell Death in Endotoxin Induced Myocardial Toxicity of Young and Aged Mice	Study the age dependent mechanism behind susceptibility to sepsis (40)	2016	China
Hermans G, et al.	Effect of tolerating macronutrient deficit on the development of intensive-care unit acquired weakness: a subanalysis of the EPaNIC trial	To evaluate whether late PN and early PN have differences in the quality control of autophagy in muscle weakness and muscle fibers (*n* = 600) (41)	2013	Belgium
Zhang J, et al.	Effect of ketogenic diet on exercise tolerance and transcriptome of gastrocnemius in mice	The impact of KD on muscle strength and exercise endurance (42)	2023	China
Weckx R, et al.	Efficacy and safety of ketone ester infusion to prevent muscle weakness in a mouse model of sepsis-induced critical illness	Study on whether ketoester-β-hydroxybutyrate is a safer alternative for treating SAW (*n* = 376) (43)	2022	Belgium
White RH, et al.	Hormonal and metabolic responses to glucose infusion in sepsis studied by the hyperglycemic glucose clamp technique	Initial hormonal and metabolic responses of sepsis patients to glucose under controlled conditions (*n* = 23) (44)	1987	Australia
Giovannini I, et al.	Respiratory quotient and patterns of substrate utilization in human sepsis and trauma	The lower mean respiratory quotient of septics indicates that they depend generally more than nonseptic trauma patients on fat as an energy substrate and confirms a previously obtained evidence of limited hepatic lipogenesis in sepsis (*n* = 99) (45)	1983	Italy
Langley RJ, et al.	An integrated clinico-metabolomic model improves prediction of death in sepsis	Different molecular processes between surviving and deceased sepsis patients may allow for the deployment of more appropriate treatment methods (46)	2013	America
Gill SK, et al.	Increased airway glucose increases airway bacterial load in hyperglycemia	Does the increase in airway glucose caused by hyperglycemia lead to an increase in bacterial load 1 (47)	2016	Britain
Darby AM, et al.	High sugar diets can increase susceptibility to bacterial infection in *Drosophila melanogaster*	Which aspects of host and pathogen physiology are impacted by diet to influence infection dynamics (48)	2024	America
Petronilho F, et al.	Obesity Exacerbates Sepsis-Induced Oxidative Damage in Organs	Assuming obesity exacerbates oxidative damage to peripheral organs in rats receiving sepsis animal models (49)	2016	Brazil
Beylot M, et al.	Regulation of ketone body flux in septic patients	To assess the effect of sepsis on ketone body (KB) kinetics in humans (*n* = 19) (50)	1989	France
Rahmel T, et al.	An open-label, randomized controlled trial to assess a ketogenic diet in critically ill patients with sepsis	Assessing whether KD can induce stable ketosis in critically ill sepsis patients (*n* = 40) (51)	2024	Germany
Cabo R, et al.	Effects of Intermittent Fasting on Health, Aging, and Disease	Discussing clinical research protocols for testing intermittent fasting regimens in healthy individuals and patients with metabolic disorders (obesity, insulin deficiency) based on the results of preclinical studies and the latest research (52)	2019	America
Dong TA, et al.	Intermittent Fasting: A Heart Healthy Dietary Pattern?	Evaluated existing literature on the potential cardiovascular benefits of intermittent fasting and proposed directions for future research (53)	2020	America
Dulloo AG, et al.	How dieting makes some fatter: from a perspective of human body composition autoregulation	Exploring whether thin individuals have a higher risk of gaining weight due to dieting compared to obese individuals (54)	2012	Switzerland
Danial NN, et al.	How Does the Ketogenic Diet Work? Four Potential Mechanisms	Discuss the four most likely mechanisms of ketogenic diet in treating epilepsy (55)	2013	America
Goossens C, et al.	Adipose tissue protects against sepsis-induced muscle weakness in mice: from lipolysis to ketones	Exploring whether pre disease obesity prevents ICU acquired weakness due to increased availability of lipids and ketones (22)	2019	Belgium
De Bruyn A, et al.	Impact of withholding early parenteral nutrition in adult critically ill patients on ketogenesis in relation to outcome	Early parenteral nutrition enhances ketogenic activity in critically ill children (*n* = 110) (56)	2021	Belgium
Goossens C, et al.	Altered cholesterol homeostasis in critical illness-induced muscle weakness: effect of exogenous 3-hydroxybutyrate	Assuming that altered cholesterol homeostasis is associated with the development of muscle weakness induced by critical illness, and this pathway can be influenced by 3-hydroxybutyrate (*n* = 600) (57)	2021	Belgium

The most recent clinical guidelines for intensive care recommend a protein intake of 1.2–2.0 g/kg/day (30), which is associated with lower mortality rates (35). A protein intake that is higher than the recommended level is associated with negative outcomes, such as delayed recovery (33). Meta-analyses have shown that high-protein nutrition does not benefit critically ill patients (36). An observational study showed that a high protein diet only benefits the survival of non-septic patients and tends to increase mortality in septic patients (37). This is because a high-protein intake can not only lead to hypernatremia (38) but can also inhibit autophagy (39). Autophagy is a cell-regulating mechanism that clears unfolded or misfolded proteins and damaged organelles, allowing for nutrient recycling and cell survival. In a mouse model of SAW, autophagy was shown to have a protective effect that improves survival and organ function (40). Inhibition of autophagy aggravates SAW, and enhancement of autophagy ameliorates SAW (41). Thus, focusing on nitrogen balance alone to guide dietary protein interventions may delay SAW recovery. The dietary protein intake level needs to be further determined based on the type of disease, the severity of protein catabolism, the energy state, and the timing and dosage of supplementation. However, KD has been shown to enhance autophagy in skeletal muscle (42), and ketolipids can treat sepsis and prevent saws (43).

It is generally believed that carbohydrates are one of the main sources of energy for the human body and play an important role in the normal function of skeletal muscles. Patients with sepsis manifest a significant shift in overall metabolism from glucose oxidation to fatty acid oxidation (FAO), with critically ill patients having a lower level of glucose oxidation (44) and enhanced lipid metabolism (45). Significant differences in glucose metabolism and fatty acid *β*-oxidation pathways were found between sepsis survivors and non-survivors (46). The mortality rate of sepsis patients with high blood sugar is increased (47). Research has found that high carbohydrate nutrition increases the susceptibility of drosophilae to certain bacterial infections. Drosophilae with high carbohydrate nutrition may develop hyperglycemia, and some pathogens may use excess sugar in the host’s body to promote growth during infection (48). In addition, obese mice fed high carbohydrate diets can exacerbate skeletal muscle damage in sepsis (49). The commercially available enteral feeds are often very high in carbohydrates. Many intravenous drugs are administered as glucose-containing solutions. Parenteral nutrition solutions also contain large amounts of glucose. The resulting high carbohydrate load leads to an increased requirement for insulin and the inhibition of FAO and ketogenesis (50). Therefore, alternative dietary management for patients with SAW may include limiting the glycemic load or changing the dietary composition to allow sufficient fasting time to enhance FAO and ketogenesis. A study found that sepsis patients receiving KD improved hyperglycemia, achieved stable ketosis, and reduced immune dysregulation compared to those receiving high carbohydrate nutrition, and were associated with improvements in clinical indicators (51).

Intermittent fasting is a major mechanism for promoting longevity and mitigating diseases (52). It can effectively activate cytoprotective and cell-repairing pathways, including autophagy, mitochondrial biogenesis, and antioxidant defense (53). However, prolonged fasting ultimately comes at the cost of weight loss (54). Therefore, the beneficial pathways facilitated by fasting can be similarly activated through modified nutritional strategies, such as ketone supplementation (55). The finding that obese patients with sepsis have a lower incidence of SAW than lean patients with sepsis suggests that obesity has a protective effect on SAW, which may be attributed to the presence of KBs (22). Plasma concentrations of *β*-HB, a type of KB, are increased in pediatric mice with early childhood macronutrient deficiencies in the ICU (56). In a mouse model of sepsis, β-HB treatment was shown to ameliorate SAW (22), suggesting that it has a direct effect on muscle strength. However, the protective effects of the KD or KBs are not solely attributed to their role as energy substrates but also to their function as signaling molecules that impact regenerative pathways (57).

## Mechanism of action of the KD on SAW

### Energy metabolism

Skeletal muscles require energy for various activities (58), and ATP is required to support muscle contraction. Typically, the majority of ATP is generated through mitochondrial respiration, with anaerobic glycolysis contributing to ≤2% of total ATP production. SAW can lead to systemic hypoxia and inflammation, increased reactive oxygen species (ROS), impaired glucose and fatty acid utilization, diminished mitochondrial number and function (59, 60), increased ATP demand, and elevated adenosine monophosphate kinase (AMPK) (60) (Figure 1).

Carbohydrates are the preferred substrate for energy production under healthy conditions. However, in the critically ill population, carbohydrates can cause hyperglycemia, increase mitochondrial oxygen consumption, and increase ROS production, thereby triggering a vicious cycle of mitochondrial damage (61). The impaired translocation of glucose transporter-4 and increased insulin resistance both exacerbate the deleterious effects of hyperglycemia (62). In addition, the metabolic characteristics of SAW patients are impaired ketone production and reduced fatty acid metabolism in the liver and muscles (22). Research has found that in SAW patients, *β*-HB is preferentially absorbed by muscles and metabolized into cholesterol precursor mevalonate, rather than TCA metabolites (57). Fat, as an energy substrate, can alter muscle substrate metabolism, from using glycogen to β-HB (63). KD treatment of SAW can increase the release and metabolism of fatty acids into ketone bodies, thereby preventing and treating SAW (22).

**Figure 2:**
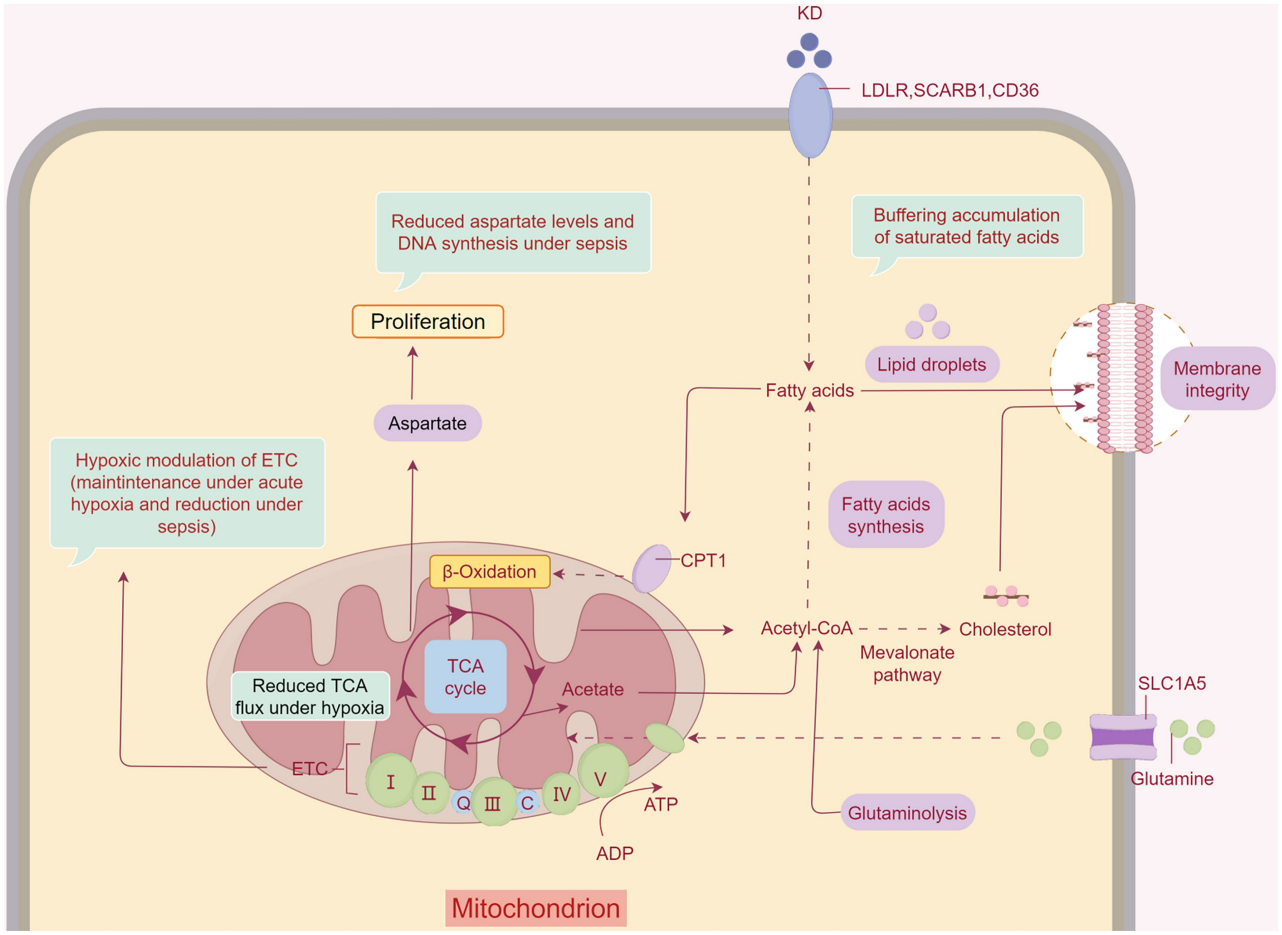
The KD ameliorates SAW by changing the energy-supplying pattern. The KD ameliorates inflammation and hypoxia in muscles during sepsis. Fatty acids provide direct relief from SAW. KBs also have therapeutic effects on SAW by affecting the tricarboxylic acid cycle and subsequently enhancing mitochondrial function. Created with Figdraw.com.

Pyruvate is transferred to mitochondria under aerobic conditions and oxidized to acetyl-CoA via the pyruvate dehydrogenase complex (PDHC) to accelerate aerobic oxidation. The PDHC activity of mononuclear cells in the peripheral blood was significantly lower in patients with sepsis than in healthy controls (64). In addition, rats with sepsis had a 70% reduction in PDHC activity and decreased acetyl-CoA production in skeletal muscle cells, resulting in hypoxia and dysfunction in skeletal muscle cells (Figure 2) (65). KD has been shown to increase pyruvate dehydrogenase levels (66). In mice fed with a KD, pyruvate was oxidatively decarboxylated to acetyl-CoA, catalyzed by pyruvate dehydrogenase (67), and the KD led to increased levels of pyruvate in skeletal muscles (68).

### Muscle signaling molecules

Wallace et al. demonstrated the efficacy of the long-term application of the KD in alleviating sarcopenia (20). The KD increases body weight and fat mass (69) but does not change body mass or muscle mass in mice (70). This may be related to its role in providing signaling molecules rather than being solely a metabolic substrate (43). The KD not only promotes the conversion of type IIb skeletal muscle fibers to type IIa fibers and increases the levels of neuromuscular junction remodeling markers, but also facilitates mitochondrial biogenesis, reduces endoplasmic reticulum stress, and enhances protein synthesis and proteasome activity (20). In addition, the KD upregulates genes related to muscle atrophy, such as *Mafbx*, *Murf1*, *Foxo3*, *Lc3b*, and *Klf15*, to reduce muscle mass, fiber cross-sectional area, and grip strength (67).

In humans, a KD can modulate tryptophan metabolism, thereby increasing mitoprotective metabolites. In addition, the anti-inflammatory fatty acids eicosatetraenoic acid and docosahexaenoic acid may be increased. During a KD, there may be increased utilization of carnitine and changes in the tryptophan pathway with decreased quinolinic acid and increased kynurenic acid concentrations. Kynurenic acid is thought to have protective effects on mitochondrial respiration, whereas accumulation of quinolinic acid is associated with mitochondrial dysfunction (71).

Animal studies have found that the administration of *β*-HB in combination with parenteral nutrition reduces muscle weakness (43). Moreover, β-HB was shown to increase the levels of muscle regeneration markers and decrease the expression of histone deacetylase (HDAC) 4 and 5, which are inhibitors of the muscle regeneration pathways (43). β-OHB injections in healthy adults reduce leucine oxidation and enhance the incorporation of leucine into the skeletal muscles (72). In addition, supplementing SAW mice with β-HB increased markers of early muscle regeneration and reduced the expression of class IIa histone deacetylases (22), which are known factors that inhibit the regeneration pathway by suppressing myocyte enhancer factor 2 (73).

### Immunity and the gut microbiota

Immunometabolism plays a critical role in host defense (74). The metabolic determinants of the host response are complex and specific to the types of infection and immune cells involved. A patient’s nutritional status alters their gut microbiota and intestinal integrity, affecting inflammation and their host response to sepsis (75). Therefore, it is necessary to consider the balance of these potentially conflicting metabolic demands when designing nutritional interventions.

Sepsis changes the gut microbiota and metabolites. Specifically, the beneficial microorganisms are replaced by the pathogenic ones (76). The gut microbiota is closely intertwined with the host immune system (77) and serves an essential function in host metabolism and resistance against pathogen colonization (78). The disturbance of the gut microbiota occurs in a variety of diseases, including infections and sepsis (79). The gut microbiota may potentially link the immune system and sepsis (80). Recent preclinical evidence has suggested a complex crosstalk between the gut microbial environment and skeletal muscles (81). Lack of gut microbiota found in animal studies can lead to muscle mass loss (82). An increase in the Rikenellaceae of elderly mice was found to be associated with muscle atrophy due to the presence of Riederia bacteria (83). In addition, compared with healthy adult rats, muscle atrophy rats have a higher ratio of Sutterella to Barnesiella, reduced size of gastrocnemius and triceps, and altered immune function (84). Luckily, the KD can maintain gut microbiota homeostasis. For instance, it increases the beneficial gut microbiota, such as *Akkermansia muciniphila* and *Lactobacillus* (85). Another study in mice and humans found that a KD led to a reduction in *Bifidobacterium* and a decrease in pro-inflammatory Th17 cells in the gut and visceral fat (86). In addition, a KD maintains immune cell homeostasis and promotes cell survival after bacterial infections (86). For instance, it promotes macrophage polarization (87), resulting in a 50% increase in M2-type macrophages and a 50% decrease in M1-type macrophages (88).

### Inflammation and gut microbiota

Persistent inflammation is one of the main mechanisms leading to the loss of skeletal muscle mass and function. In the clinical-translational setting, the fundamental importance of ketogenic metabolism for human T cells has already been demonstrated in the context of critically ill COVID-19 patients and, in particular, the improvement of T cell immune metabolism by ketone bodies has been demonstrated in compromised T cells of intensive care patients (89). Due to the profound change in macronutrient composition, a ketogenic diet also has significant effects on the cerebrospinal fluid metabolome, which indirectly exerts additional modulating influences on immune cell populations (90).

**Figure 3:**
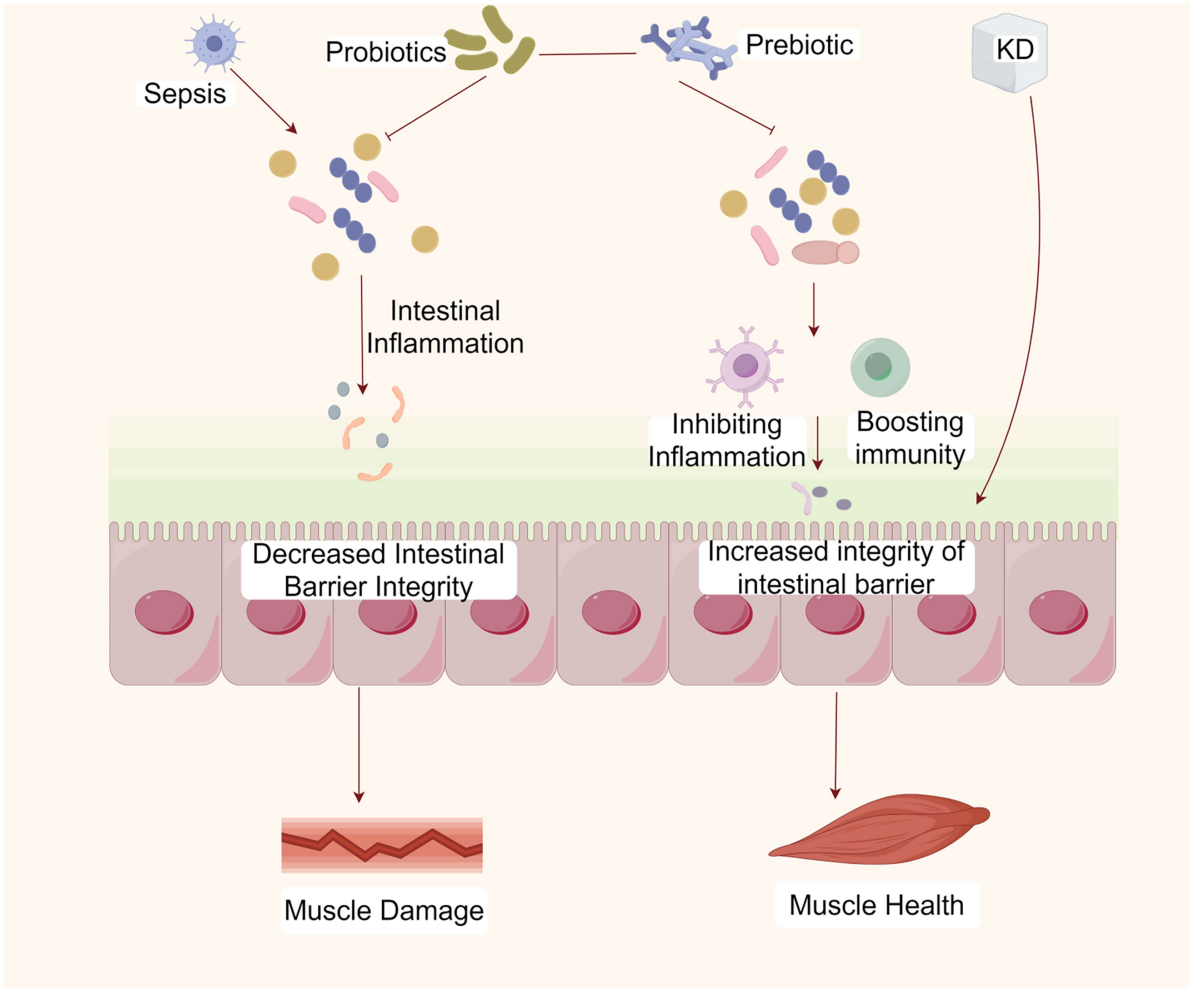
The KD ameliorates SAW by maintaining gut microbiota homeostasis. The KD can maintain the homeostasis of the gut microbiota, the integrity of the intestinal barrier, and immune homeostasis, thereby inhibiting inflammation and ameliorating SAW. Created with Figdraw.com.

Intestinal disorders and the loss of gut microbiota diversity impair the integrity of the intestinal barrier and can allow harmful microbial products, such as lipopolysaccharides, to enter the bloodstream, leading to systemic inflammation and metabolic disorders, which can weaken muscle function and reduce muscle mass (91) (Figure 3). The gut microbiota promotes metabolic homeostasis and immune function by strengthening the intestinal barrier. The loss of gut microbiota diseases and diversity can damage the integrity of the intestinal barrier, allowing harmful microbial products such as lipopolysaccharides (LPS) to enter the bloodstream. These harmful substances can cause systemic inflammation, leading to metabolic disorders and decreased muscle function and quality (91). When the intestinal epithelial barrier function is impaired, under lipopolysaccharide stimulation, nuclear factor kappa B (NF-kB) translocates from the cytoplasm to the nucleus, and stimulates dendritic cells and macrophages to produce pro-inflammatory cytokines and mediators, such as cyclooxygenase-2, TNF-*α*, inducible nitric oxide synthase, and IL-6, which regulate intestinal and systemic inflammation (92). And TNF-α regulates the activation of NF-kB signaling pathway by expressing atrophy related genes, and promotes protein degradation through transcription of ubiquitin proteasome E3 ligase (93). It was reported that the expression level of *SOCS3*, the target gene of IL-6, was elevated in the skeletal muscles of patients with SAW and that L-6 mediates sepsis-induced muscle atrophy through the gp130/JAK2/STAT3 pathway (94). Research has found that KD not only alleviates TNF α-induced apoptosis and inflammation of intestinal cells (95), but also reduces the increase in IL-6 levels caused by gut microbiota disorders (96).

Insulin resistance and reduction in skeletal muscles are associated with increased Toll-like receptor 4 (TLR4) expression and signaling in an aging model, which may be due to the development of secondary endotoxemia (97). Activation of the TLR4 signaling pathway results in significant increases in the NF-κB protein level and the phosphorylation level of c-Jun N-terminal kinase (97). The TLR4 signaling pathway can also induce a systemic inflammatory response by upregulating pro-inflammatory cytokine (IL-6 and TNF-α) levels through a cascade reaction (98). These inflammatory cytokines are involved in regulating muscle atrophy. Inhibition of the production of these inflammatory cytokines by modulating the gut microbiota can alleviate muscle atrophy (99). Many studies have now confirmed that a KD improves the inflammatory environment by regulating the gut microbiota (Table 2). A KD upregulates antioxidant and anti-inflammatory pathways (100). For example, Ketones are anti-inflammatory and can suppress chronic low-grade inflammation by inhibiting the NLRP3 inflammasome (101). At the same time, however, the antiviral immune response of gamma/delta T cells is enhanced by a ketogenic diet, and the use of ketone bodies has been postulated as an antiviral therapy option (102). And *β*-HB inhibits the formation of NLRP3 inflammatory vesicles and prevents the release of pro-inflammatory cytokines (103).

**Table 2:** Effects of ketogenic diet on immunity.

Disease	Intestinal flora	Inflammation	References
Cognitive impairment (CI)	Changed the gut microbiota	Th1 cells ↓	Olson et al. (104)
Neuroinflammation	Firmicutes and Proteobacteria ↑ Bacteroidetes ↓	TNF-*α*, IL-1*β*, and IL-6 mRNA ↓	Li et al. (105)
Parkinson’s disease (PD)	Bifidobacterium↓	Th17 cells ↓	Ang et al. (106)
Parkinson’s disease (PD)	Citrobacter, Desulfovibrio, Lactobacillus, and Ruminococcus ↓	TNF-α, IL-1β, and IL-6 ↓	Jiang et al. (107)
Herpes simplex virus type 1 (HSV-1), Infection-associated herpes simplex encephalitis (HSE)	Lactobacillus and *Akkermansia muciniphila* ↑	*TNFα*, *IL-6,* and *NOS2* ↓	Shan et al. (108)
Alzheimer’s disease (AD)	Proteobacteria, Enterobacteriales ↑	TNF-α and IL-1β mRNA ↓	Park et al. (109)
Drug-resistant epilepsy	Actinobacteria, Bifidobacteria ↑, and Proteobacteria ↓	Anti-inflammatory	Rohwer et al. (110)
Acute pancreatitis	Enterobacterales ↑	IL-1α, IFN-*γ* ↓	Xia et al. (111)
Drug-resistant epilepsy	Bifidobacteria ↓	IL-17A, IL-17C, TNF, IL-12B, IL-18R1, and GDNF ↓	Dahlin et al. (112)
Sepsis	Intestinal epithelial cells ↓	IL-1β, IL-6, and TNF-α ↑	Quan et al. (113)
Critical illnesses	Bacilli, Lactobacillales ↑	Immune ↓	Xu et al. (114)
Sarcopenia	Gut dysbiosis	Immune response ↓ and promoting inflammation	Nardone et al. (115)

## Conclusion and outlook

SAW involves unique metabolic entities compared to other critical illnesses. Therefore, optimal metabolic and nutritional management strategies may differ between critically ill patients with SAW and non-SAW. Increasing evidence suggests that transitioning from a carbohydrate-centric metabolism to one that prioritizes lipid metabolism may offer protective effects against SAW. This involves mechanisms like maintaining mitochondrial homeostasis, producing anti-inflammatory effects, and regulating immune homeostasis and gut microbiota. However, the benefits of a KD on SAW remain controversial and need validation with further clinical and basic studies. In addition, this article is a narrative review and lacks a systematic retrieval strategy in the literature collection process, requiring more systematic evidence summary and analysis.
